# Coupled Information Diffusion–Pest Dynamics Models Predict Delayed Benefits of Farmer Cooperation in Pest Management Programs

**DOI:** 10.1371/journal.pcbi.1002222

**Published:** 2011-10-13

**Authors:** François Rebaudo, Olivier Dangles

**Affiliations:** 1 UR 072, Institut de Recherche pour le Développement (IRD), Gif-sur-Yvette, France; 2 UPR 9034, Centre National de la Recherche Scientifique (CNRS), Gif-sur-Yvette, France; 3 Université Paris-Sud 11, Orsay, France; 4 Facultad de Ciencias Exactas y Naturales, Laboratorio de Entomología, Pontificia Universidad Católica del Ecuador (PUCE), Quito, Ecuador; Pennsylvania State University, United States of America

## Abstract

Worldwide, the theory and practice of agricultural extension system have been dominated for almost half a century by Rogers' “diffusion of innovation theory”. In particular, the success of integrated pest management (IPM) extension programs depends on the effectiveness of IPM information diffusion from trained farmers to other farmers, an important assumption which underpins funding from development organizations. Here we developed an innovative approach through an agent-based model (ABM) combining social (diffusion theory) and biological (pest population dynamics) models to study the role of cooperation among small-scale farmers to share IPM information for controlling an invasive pest. The model was implemented with field data, including learning processes and control efficiency, from large scale surveys in the Ecuadorian Andes. Our results predict that although cooperation had short-term costs for individual farmers, it paid in the long run as it decreased pest infestation at the community scale. However, the slow learning process placed restrictions on the knowledge that could be generated within farmer communities over time, giving rise to natural lags in IPM diffusion and applications. We further showed that if individuals learn from others about the benefits of early prevention of new pests, then educational effort may have a sustainable long-run impact. Consistent with models of information diffusion theory, our results demonstrate how an integrated approach combining ecological and social systems would help better predict the success of IPM programs. This approach has potential beyond pest management as it could be applied to any resource management program seeking to spread innovations across populations.

## Author Summary

Food security of millions of people in the third world has faced a growing number of challenges in recent years including risks associated with emergent agricultural pests. Worldwide, the promotion of integrated pest management practices has been heavily promoted through participative methodologies relying on farmer cooperation to share pest control information. Recent studies have put into doubt the efficiency of such methodologies evoking our poor knowledge of farmers' perceptions, behavioral heterogeneity, and complex interaction with pest dynamics. While pest management programs have a larger place than ever on the international policy agenda, the debate concerning their efficiency at large scales has remained unresolved. Here, we developed an innovative modeling approach coupling pest control information diffusion and pest population dynamics to study the role of cooperation among farmers to share the information. We found that the slow learning process placed restrictions on the knowledge that could be generated within farmer communities over time, giving rise to natural lags in pest control diffusion and applications. However, our model also predicts that if individuals learn from others about the benefits of early prevention of invasive pests, then a temporary educational effort may have a sustainable long-run impact.

## Introduction

In view of the growing number of challenges related to controlling agricultural pests, the promotion of Integrated Pest Management practices (IPM; a range of methods used for responsible pest control) has a larger place than ever on the international policy agenda [1], [2]. The participation of local communities and other stakeholders in such management processes has long been advocated as an essential step to achieve sustainable development [3]. Over the past decades, extension science has developed many types of participatory approaches towards farmers [4] to promote knowledge of agro-ecological concepts, apply IPM practices, reduce the use of pesticides and improve crop yields [5]. As budget and manpower constraints do generally not allow for direct interaction with every member of the target population, the strategy of most participative IPM programs is to train a limited number of farmers in the community who commit themselves to share the information they learn with other farmers [6]. Following Rogers' “diffusion of innovation theory” [7], the success of extension practices depends on the effectiveness of cooperation among farmers which determines IPM information diffusion from trained farmers (graduate farmers) to other farmers (exposed farmers).

Funding from international development organizations often relies on the important, but poorly studied, assumption that farmers cooperate with their peers, neighbors, or friends [8]. Increasing our understanding of farmers' cooperation theory and practice is a timely issue as field-level interactions among small-scale farmers are increasingly limited in a world of intense social reorganizations associated with land distribution, privatization of ownership, and market-oriented society [9].

A collective action problem that requires farmers to cooperate in information diffusion is exemplified by invasive pest control in fragmented agro-ecosystems [10]. If neighbors of graduate farmers do not adopt IPM measures, then the invasive pests from their fields can re-infest the graduate farmers' fields even if they apply IPM principles [11]. Moreover, in the case of emergent invasive species, farmers cannot rely on preexisting local knowledge, which makes them even more dependent on externally based experience. In farmer communities, IPM for invasive species is therefore characterized by a conflict of interest between individual and group benefit leading to cooperation dilemma [12], [13]. On the one hand, cooperation by graduate farmers to share IPM information is expected, in the end, to benefit the whole community of farmers (including themselves) by an area-wide suppression of the pest. On the other hand, under the assumption that graduate farmers want to prioritize control in their fields instead of training other farmers, theory predicts that individuals might have little incentive to cooperate and will not contribute to the public good [12]. Both types of behaviors have been classically observed in a wide array of agricultural situations [1]. In the specific case of IPM, farmers' decisions about whether to disseminate or not pest control practices will be closely dependent on pest infestation levels in their own field [1]. This means that farmers' dilemma to train others or not will be tightly linked to pest dynamics at the landscape level, itself depending on landscape characteristics, pest ecology and control behaviors of other famers. Exploring the relative merits of helping others vs. self interest in IPM information diffusion therefore requires the coupling of ecological and sociological models, an approach which has, to our knowledge, never been performed in the context of IPM.

**Figure 1:**
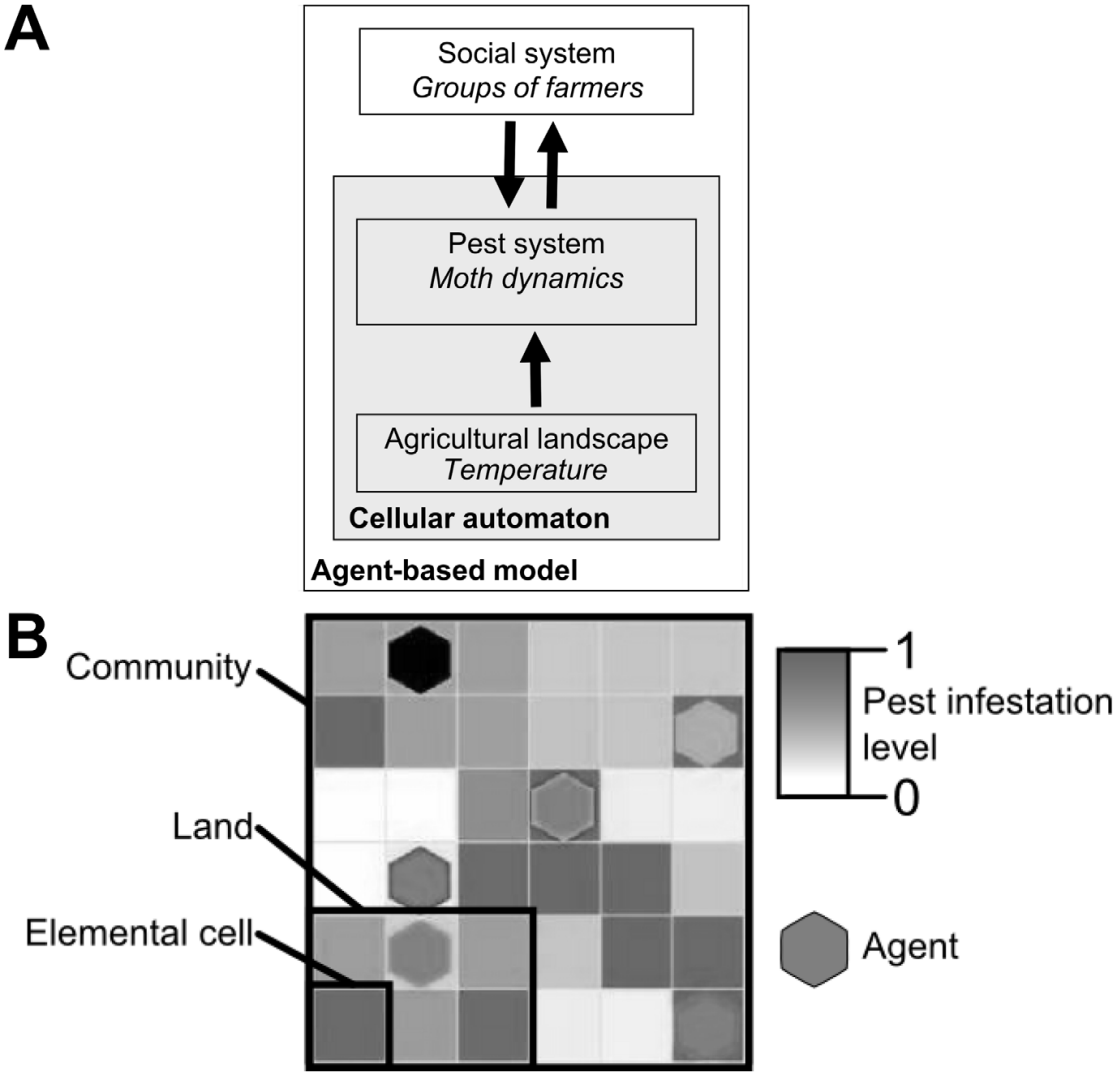
Model schematization. A. The cellular automaton (pest population dynamics sub-model driven by temperature) is coupled to an agent-based model, made by agents controlling the pest and exchanging pest management information as a function of infestation levels in their land. B. Representation of the model where the community consists of 36 cells, divided into 6 lands of 6 elemental cells. Each cell sizes 500×500 m. One agent (represented by an hexagon) is assigned to each land. The green gradient indicates pest infestation level, from no presence in white to the carrying capacity of each cell in dark green. Each agent interacts both with pest (control) and other agents of their community (pest management information exchange).

The objective of our study was to develop a methodological framework to explore the relevance of participative IPM extension programs for pest control. We carried out these investigations in the context of an IPM program launched to help small scale farmers facing the arrival of an invasive insect pest, the potato tuber moth (*Tecia solanivora* Povolny) in the Ecuadorian Andes [14]. This region was highly relevant for our study as there is a long history of social reciprocity in the Andes that extends to pre-Incan times and has been one of the keystones for why farmers have been able to successfully farm for centuries in such harsh conditions [15]. We then built an agent-based model (ABM, [16], [17]) merging a spatially explicit pest population dynamic model through a cellular automaton (CA) with a field-based multi-agent system describing farmer features and behaviors (Fig. 1A). The global output of our ABM was determined from pest–landscape interactions, pest-farmer interactions, and inter-farmer interactions. To mimic real-world patterns of farmer behaviors as closely as possible, our ABM was implemented with field data, including learning processes and control efficiency, from large scale surveys from c.a. 300 farmer households in the Ecuadorian Andes. In our model, the agricultural landscape was modeled as a lattice composed of cells that represented various land plots of groups of farmers (hereafter named agents) within the same community (in total, 6 neighbor agents in the same community representing about 220 people, Fig. 1B). Pest dynamics was driven by the intrinsic population growth, migration, and pest control practiced by agents depending on their IPM knowledge. Under our IPM program, one agent was trained to control pest infestation in his fields. In return, this graduate agent was required to diffuse the IPM information to other agents so that they can increase their IPM knowledge and implement efficient practices. Agent decision to diffuse the information to others mainly depended on pest infestation level in his fields but also on social and economic factors included in the diffusion process of IPM information among farmers. Therefore, pest control at the community level was modeled as emerging from IPM information acquired by one graduate agent and spreading through exposed agents (see Text S1).

We believe that the relevance of our study stands in two main points. First, recent works on collective actions of IPM diffusion have reported that because behaviors and perceptions towards new information and technology can vary widely among farmers, farmers' behavioral heterogeneity is a key issue to understand and predict the success of pest control information diffusion throughout the community, and therefore the success of the IPM program at a large scale [14], [18]. In this context, ABMs may reveal ideal tools to better understand and predict the sustainable development of farmers' control practices [19]–[21] as they allow simulating the actions and interactions of autonomous agents (either individual or collective entities such as organizations or groups of farmers) with a view to assessing their effects on the system as a whole. Using ABM therefore allows integrating behavioral complexity of farmers and performing theoretical experiments (e.g., varying the level of farmer cooperation) which could not be performed in the real world (for time, ethical or financial reasons). Although ABM have increasingly been applied to physical, biological, medical, social, and economic problems [22], [23], [16] it has been, to our knowledge, completely disregarded by IPM theory and practice. Second, our study proposes an innovative computational framework merging recent advances in contagion-like model of knowledge diffusion through human populations [24], [25] and coupled land management models with spatially explicit species spread models (see papers presented at LandMod 2010 or Global Land Project 2010). Such a framework combining two approaches which developed in relative independence likely has potential beyond pest management as it could be applied to any resource management program seeking to spread innovations across populations.

## Results

**Figure 2:**
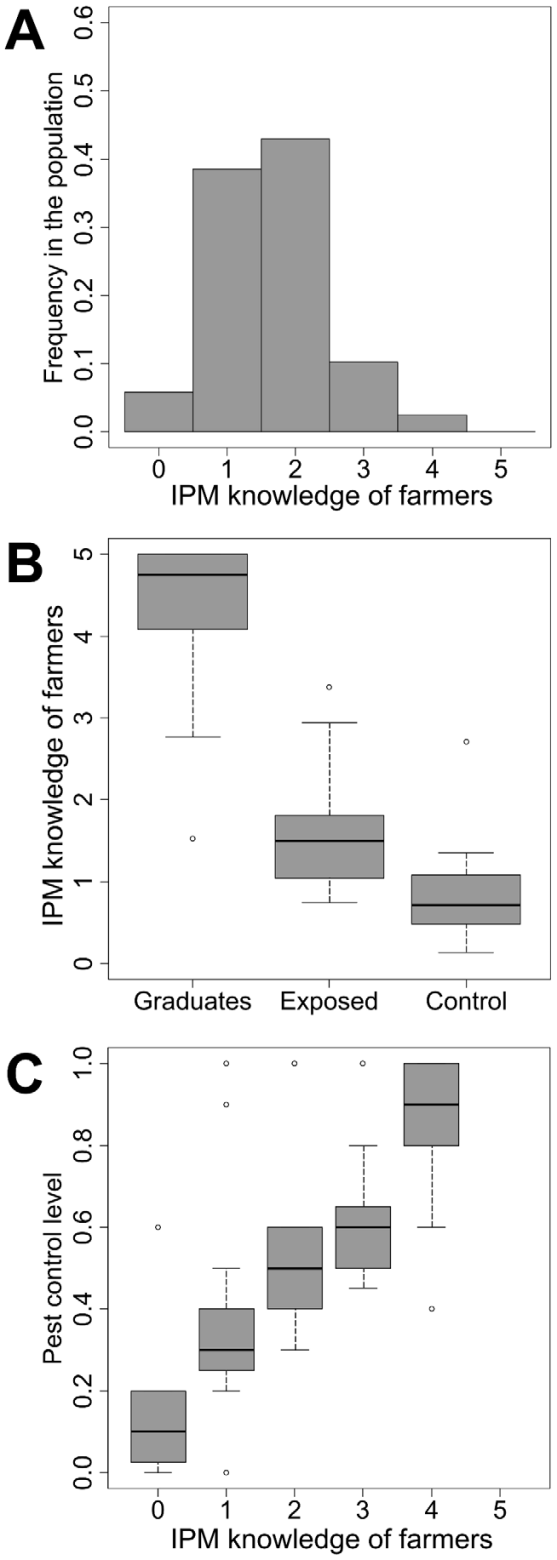
Field data. A. Distribution of IPM knowledge of farmers (n = 293 inquests) B. Efficiency of learning process between graduates and exposed farmers (n = 85). C. Relationship between IPM knowledge of farmers and pest control (n = 83 households) (linear model; R^2^ = 0.51, P<0.001).

The field survey revealed that, at the beginning of our program, a majority of farmers (87%) had a low IPM knowledge (score ranging between 0 and 2) regarding potato moth control (Fig. 2A). Our data further showed that although this knowledge could be greatly increased through training (graduate farmers reached an IPM knowledge of 4.39±0.61), those skills were not easily diffused to exposed farmers by informal training sessions (Fig. 2B). After having graduate farmers shared information with exposed farmers the mean knowledge score of the 64 surveyed exposed farmers increased only slightly when compared to control, from 0.96±0.80 to 1.65±0.53 (Student t-test, t = −1.717, P = 0.111). Interestingly, although moth control gradually increased with increasing IPM knowledge scores (linear model fit, R^2^ = 0.51, P<0.001), there were a few cases in which farmers with relatively high IPM knowledge had also poorly efficient pest control in their fields, probably due to contamination from neighboring fields (Fig. 2C).

**Figure 3:**
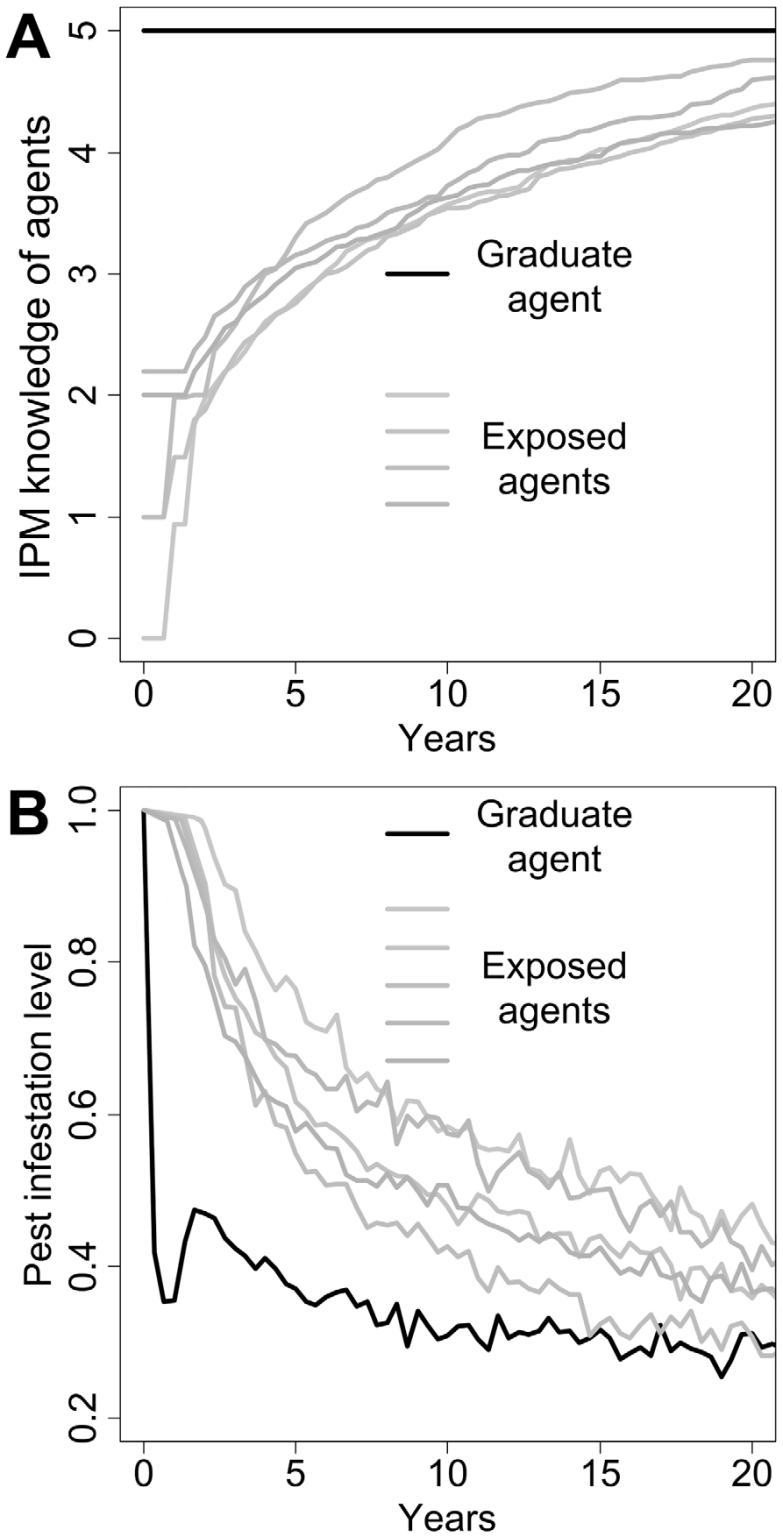
Results of ABM simulations. ABM results showing the evolution of IPM knowledge of agents (A) and pest infestation level (B) through time (mean of 100 simulations).

**Figure 4:**
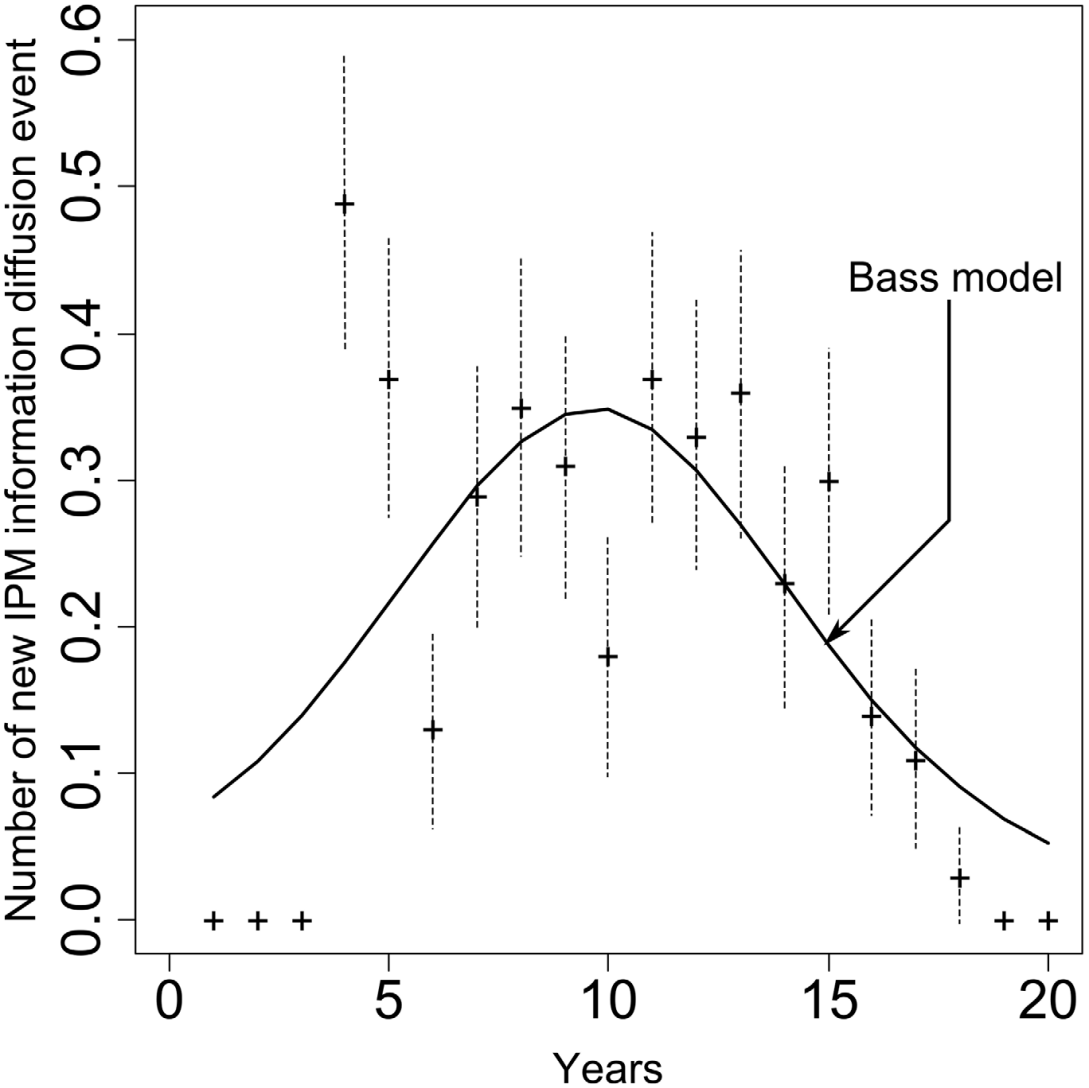
Number of new IPM information diffusion event over time fitted to the Bass model. The fit was obtained following [47] (p = 0.015±0.001, P<0.001; q = 0.296±0.011, for all parameters t≥12.67, P<0.001). Each point is the mean of 100 repetitions with confidence intervals 95% in dashed lines. The theoretical prediction curve represents the derivative of N over time.

Once the ABM was set up with these real-world data, we explored on a 20-year time scale the influence of the level of cooperation among agents (i.e. how often graduate agents did share their information with others) on pest infestation levels. Our model predicted that knowledge acquisition by exposed agents would follow a logistic regression through time (R^2^ = 0.50±0.11, P<0.05, Fig. 3A). Our simulations further predicted that both IPM knowledge diffusion and spillover after training would significantly decrease moth infestation by 60 to 70% from their initial levels (Fig. 3B). Time dedicated by graduate agents to train exposed agents instead of controlling pest had the short term consequence of increasing pest infestation in his own land (interviews with farmers revealed that training others would demand time and compromise of coordination with consequences in terms of pest control in their own field.). However, as exposed agents were being trained, graduate agents were less solicited thereby being able to dedicate more time to pest control. Importantly, the patterns of IPM information diffusion among agents predicted by our ABM was consistent with the Bass model (F-test, P<0.001, Fig. 4), a model traditionally used in diffusion of innovations [24]. The ability of our ABM to reproduce Bass model predictions therefore provided a validation of the correctness of information adoption patterns among agents, mainly through internal (“word-of-mouth”) influences.

**Figure 5:**
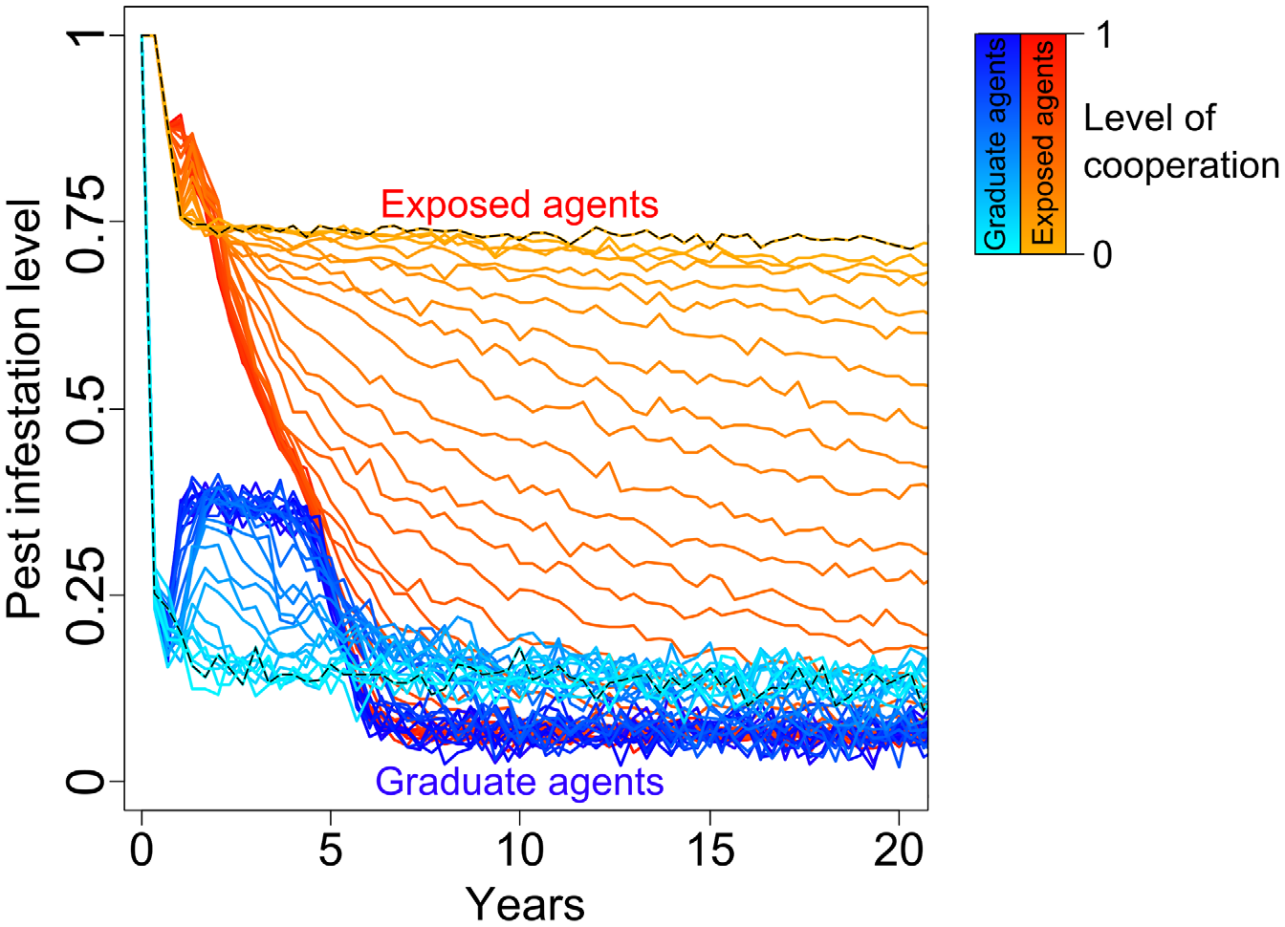
Influence of cooperation among agents on pest infestation in fields of exposed (red) and graduate (blue) agents.

Results of our simulation of the effect of farmer's cooperation level on pest control showed that within the first 6–7 years, pest infestation levels in both graduate and exposed agents' lands remained higher than those expected in the lands of a non-cooperating agent, whatever the cooperation levels. After 6–7 years, cooperating graduate agents had lower pest infestation level than non-cooperating ones, and therefore received the benefit of cooperating. Finally, for high levels of cooperation among agents (>0.5), our model predicted that after 6–7 years, pest infestation levels at the scale of the entire community (i.e. in all lands of agents) would be lower than levels expected in the fields of a non-cooperating graduate agent. The benefit of cooperation had therefore scaled up at the level of the whole community of agents (Fig. 5).

## Discussion

Since the emergence of the concept of knowledge based economy [26], the analysis of information diffusion has become a key issue to organization research [27]. Our results showed that the slow IPM learning process measured in Andean farmer communities placed restrictions on the amount of information that could be diffused within the community over time, giving rise to natural lags in IPM applications. This reinforces the view that IPM outcome at the community level will be achieved on a relatively long-term scale for the farmer, a feature which may be common to many agriculture programs. In an influential study that spawned an enormous diffusion of literature in rural sociology, [28], estimated that it took 14 years before hybrid seed corn was completely adopted in two Iowa communities. Rogers [7] also reported slow adoption in crop protection management in the Colombian Andes and Berger [21] showed that behavioral heterogeneity among Chilean farmers, delayed for almost 10 years the use of new irrigation methods. In our study, the six year delay in benefits of cooperation was mainly due to the limited spread of IPM information from graduate to exposed farmers which itself may have been a consequence of high IPM knowledge heterogeneity among farmers. Information is indeed expected to flow less smoothly in a heterogeneous population, particularly when the performance of new practices is sensitive to imperfectly transmitted information [29].

Our simulations also showed that there were short-term costs for the diffusion of IPM information resulting from our assumption that farmers cannot control pests in their own fields when they share IMP information with other farmers. Indeed, “lack of time” is a common motive invoked by farmers when they are questioned why they do not share IPM practices they learned with neighboring farmers [30]. As farmers often believe that there is a trade-off between diffusing and practicing IPM information, we think that an important outcome of our study was to show that, even if such a trade-off is included in the model, cooperating farmers would still benefit from IPM information diffusion in the long run. It is also likely that, in some cases, farmers may practice and diffuse new information simultaneously [1]. Cooperating farmers would then not suffer from short-term costs, potentially increasing their cooperation will, thereby speeding up information transfer throughout the community.

Obviously, our modeling approach made a series of simplifications which may be important to consider. For example, farmers usually tend to make high contributions initially but over time contributions dwindle to low levels. Many people are conditional cooperators, who in principle are willing to cooperate if others do so as well, but get frustrated if others do not pull their weight [31]. In agricultural systems personal networks, where trusted people (prestigious individuals, people of authority or holding otherwise vested power and influence) often play a key role in decision making, are difficult to integrate into models due to their dynamic, multi-directional, and non-symmetric nature [32]. Moreover the spread of behaviors may arise from the spread of social norms or from other psychosocial processes, such as various types of innate mimicry [33]. A recent study has shown that cooperative behaviors can cascade in human social networks even when people interact with strangers or when reciprocity is not possible; people simply mimic the behavior they observe, and this mimicking can cause behaviors to spread from person to person to person [34]. In this case, the rate of diffusion is largely dependent upon the knowledge (i.e., relative advantage, compatibility within the social setting, observability, and simplicity). Finally, another limitation may arise from the use of a behavioral reciprocity model. Theoretically, the adoption of IPM cooperative behavior among farmers could be favored as the reciprocated benefit outweighed the immediate cost [27]. However, in practice, the delay between the cost of a cooperative act and the benefit of reciprocated cooperation (from 7 to 20 years for graduate agents in our study) would introduce a number of cognitive challenges. For example, temporal discounting (for example devaluing of future rewards in the case of shift in crop type produced), often results in a preference for smaller, immediate rewards over larger, delayed rewards [35]. Variation in human discounting and cooperation validate the view that a preference for immediate rewards may inhibit reciprocity [35].

Despite these limits the ability of our model to capture real-world patterns of pest control (Fig. S5 in Text S1) and information diffusion (Fig. 4) indicates that our findings may yield important insights for IPM science and policies. First, IPM programs worldwide are confronting the reality of increasingly subdivided habitats managed as smaller areas, reducing the likelihood that pest population will be controlled, thereby requiring higher levels of cooperation among farmers [10]. We showed that when farmers make control decisions based on lower levels of damages occurring on their own land, they can increase information spread and the speed with which the whole community can control pest populations. Second, our study stresses the need to develop a comprehensive and empirically-based framework for linking the social and ecological disciplines across space and time [19]. In our model, predictions of the coupled dynamic of pests and farmer behavior show the evidence that farmer to farmer training can help the broader community control pest infestation in the long term. Third, as institutions increasingly seek to help communities sustainably providing local public goods themselves rather than depend on external assistance, the idea that development projects should aim at financial sustainability through local cooperative actions has had tremendous influence on funders. Our study shows that sustainable approaches to providing local public goods concerning invasive pest control would be possible despite a challenging delay between the cost of a communal act and the benefit of reciprocated cooperation. However, if individuals learn from others about the benefits of early prevention of invasive pests (i.e. cooperation takes from low levels of pest populations), then a temporary educational effort may have a sustainable long-run impact.

## Materials and Methods

### Study area

We addressed the issue of the importance of farmer cooperation in invasive pest management in the socio-agricultural system of the Ecuadorian highlands where potatoes (Solanum tuberosum L), are a major staple [36]. In 1996 a new pest, *T. solanivora*, invaded the country attacking potato tubers in the field and in storage and becoming one of the most damaging crop pests in the region [37]. Under the climatic conditions of the Ecuadorian highlands (*sierra*) potatoes are grown at any time of the year between elevations of 2400 m and 3800 m elevation [38]. The agricultural landscape of the highlands is made up of a mosaic of small potato fields (<1 ha) at various stages of maturation in which potato moths are active all year round. IPM programs have been implemented for about 10 years by the INIAP (Ecuador's National Institute for Agronomy Research) and the CIP (International Potato Center), through the Farmer Field School methodology [39]. In the North Andean region, collaborative work in the form of “*mingas*” and “*Aynis*” is necessary among small groups of farmers in order to realize hard tasks like sowing or harvesting. These labor force exchanges, despite of being very hierarchical, share common practices [40]–[42].

### Model overview

We built a representation of socio-agronomical landscapes of the central Andes at an altitude of 3000 m, which corresponds to the zone where most farmers cultivate potato. This landscape comprised three key elements: the socio-agricultural landscape, the potato moth population, and the groups of farmers (Fig. 1B). First, characteristics of the socio-agricultural landscape were set up using data from published field surveys: 1) the median community size in the study area was about 150 people [14] which roughly corresponded to 6 household units (i.e. a group of fields cultivated by one group of farmers). 2) The size of elemental cells was set up to 500 m×500 m in order to accurately model pest dispersion among cells with regards to insect's flight capability [43]. 3) Seasonal variability in climatic features (both temperature and rainfall) for each cell was obtained using the Worldclim data set [44].

Second, potato moth dynamics were simulated through a cellular automaton (CA) recently developed by our team [43]. Briefly, the CA is spatially explicit, stage-structured, and based on biological and ecological rules derived from field and laboratory data for *T. solanivora*'s physiological responses to climate (temperature and rainfall). Main processes include moth survival (climate dependent), dispersal to neighbor cells through diffusion processes (density dependent), and reproduction (climate dependent) (see Fig. S1 in Text S1). In each time step (equivalent to one moth generation, about 2 months) the infestation grows and spread over household units. A Mathematical presentation of the underlying principles of the pest model, along with general results identifying the important simulation details and their consequences, are given in [45].

Third, to transfer the pest model into an ABM we populated the agricultural landscape with artificial agents acting individually upon pest dynamics (see Fig. 1A and Appendix for a complete description of the model structure). Briefly, each agent represented a group of farmers and was set with a behavioral model that guided his or her decisions. Potato moth control at the community level was modeled as emerging from IPM information spreading through agents that composed the community. The ability to learn IPM recommendations was considered as an adaptive trait that indirectly contributed to agent's fitness by improving their capability of controlling pest populations (and therefore assuring their crop production). Agents with different IPM knowledge interacted directly with each other to exchange information (agents with less information learned from other agents). We used a reciprocity model for cooperation in which agents paid a short term cost of cooperation for the future benefit of a community member's reciprocated cooperation [35]. Agents indeed perform multiple roles which constrict the amount of time and energy they may allot to any single activity. They perceived and controlled pest infestation levels in their field depending on their IPM knowledge (see below and Protocol S1, S2).

### Setting up agent behavior rules with field survey data

To explore the profitability of our IPM program as a function of the coupled dynamics of agent behaviors (and learning spillover) and pest population, we needed three pieces of field information: 1) the initial IPM knowledge of each agent in the community, 2) the relationship between IPM knowledge and pest control, and 3) the efficiency of IPM information diffusion between graduate and exposed agents (including a wide range of social factors influencing innovation diffusion). We acquired these data through a farm-level empirical survey from nationally representative samples of farmers in rural Highland Ecuador. Our database was obtained through a three-year household survey conducted in 2006–2008 in four provinces of the Ecuadorian highlands (Bolivar, Tungurahua, Cotopaxi, and Chimborazo) using standard household survey techniques [46]. Survey zones had not been covered by any educational program regarding potato moth management. In total, 293 potato grower families from about 100 different communities were interviewed, gathering data on IPM knowledge in communities and pest control. The efficiency of IPM learning and dissemination processes was assessed through farmer field schools as described in details by [30]. Briefly in each target community, we first performed a baseline study of IPM knowledge for as many community members as possible. Farmers interested in IPM extension were then trained through FFS procedures during eight one-day sessions over the duration of potato crop cycle (about 4 months). Each graduate farmer committed himself in training at least five other farmers. Informal discussion with trained framers revealed that the amount of time they dedicated in training other farmers varied greatly, between several hours to several days. Exposed farmers were then interviewed to measure their IPM knowledge and the efficiency of the IPM information diffusion process.

### Cooperation rules among agents and ABM simulations

In each community, the IPM knowledge of agents were set up according to the frequency distribution presented in Fig. 2A (one agent with a score of 0, two with a score of 1, two with a score of 2, and one with a score of 3). We then increased the knowledge of the agent with a score of 3 to a score of 5 as if it had participated in a FFS (see Fig. 2B). This agent became the graduate agent of the community. According to FFS recommendations, this agent (in the case he or she was eager to cooperate) shared his information with exposed agents of his community (defined as an agent with a lower IPM knowledge). Once other exposed agents achieved, in turn, a higher IPM knowledge, they could also share their information with neighbor agents. An agent could share information with only one agent with a lower IPM knowledge (during this time the farmer could not control pest in his fields). When not sharing their information each agent was able to control pest in his field with an efficiency which depended on their IPM knowledge (following Fig. 2C). Again, the pest level in each cell was driven by both intrinsic population growth and diffusion from neighbor cells (see above).

Once the ABM was set up and sensitivity analysis performed (Fig. S2–S4 in Text S1), we further explored how agents' level of cooperation (i.e. how available agents were to share their information with others) would influence the benefits of our IPM program at both individual farmer and community levels. Because decision of poor farmers to cooperate for crop protection is likely to be driven by self-interest rather than altruism [14], [15], we assumed that farmers would be more prone to cooperate in IPM information diffusion when they perceive that a pest represents a danger for themselves. In our model, varying levels of cooperation were obtained by changing the pest infestation level that triggered a control action by agents (see Text S1). Each simulation was repeated 100 times over 120 time steps (i.e. about 20 years) and pest infestation levels were given for exposed agents, graduate agents, and the whole farmer community.

## Supporting Information

Protocol S1 **Source code of the model.** The source code was written using CORMAS (March 2008 release) developed with the non-commercial version of VisualWorks® from *Cincom Systems*.
(TXT)

Protocol S2 **Source code of the model (additional environmental file).**
(TXT)

Text S1 **Extended materials and methods.** This document includes empirical field data, a model description and model analysis: verification and validation.
(DOC)
