# Supplementary material for: Coupled Information Diffusion–Pest Dynamics Models Predict Delayed Benefits of Farmer Cooperation in Pest Management Programs
Source: PLoS Comput Biol. 2011 Oct 13;7(10):e1002222. doi: 10.1371/journal.pcbi.1002222 (PMC3192820; doi:10.1371/journal.pcbi.1002222)
Supplement: Text S1 — Extended materials and methods. This document includes empirical field data, a model description and model analysis: verification and validation. (DOC) [file pcbi.1002222.s003.doc]

**Supporting Information for**

**Coupled Information Diffusion – Pest Dynamics Models Predict Delayed Benefits of Farmer Cooperation in Pest Management Programs**

François Rebaudo, Olivier Dangles*

*To whom correspondence should be addressed. E-mail: olivier.dangles@ird.fr

This document includes

**Extended materials and methods**

1. Empirical field data

2. Model description

3. Model analysis: verification and validation

**Additional references**

**Additional figures S1 to S5**

**Additional table S1**

**Additional appendix S1**

**Extended materials and methods**

**1. Empirical data on Andean farmer behaviors**

To mimic real-world patterns of farmer behaviors as closely as possible, our ABM was built on farm-level empirical survey data from nationally representative samples of rural Ecuador. Our database was obtained through a three-year household survey conducted in 2006-2008 in four provinces of the Ecuadorian highlands (Bolivar, Tungurahua, Cotopaxi, and Chimborazo) using standard household survey techniques [1]. Survey zones had not been covered by any educational program regarding potato moth management. In total, 293 potato grower families from about 100 different communities were interviewed, gathering data on integrated pest management (IPM) knowledge in communities, levels of pest control, and the efficiency of IPM learning and diffusion processes.

To explore the profitability of IPM program as a function of the coupled dynamics of agent and pest population, we needed three pieces of field information (see Fig. 2 of the main document):

*1) Initial IPM knowledge of each agent.* To establish the initial IPM knowledge of each agent in the community, each potato grower family of our survey was asked 20 questions, 10 on basic issues (biology and ecology of the potato moth) and 10 on applied issues (potato moth management [2]). Farmer responses to these questions allowed us to establish IPM knowledge scores (ranging from 0 to 5). These data were implemented in our ABM to set up the baseline proportion of the different knowledge scores for the agents.

*2) Relationship between IPM knowledge and pest control.* For 83 of the 293 interviewed households, we quantified the effect of natural *T. solanivora* larvae infestation on potato plants at harvest time as a proxy of pest control. We determined damage levels for 10 plants in the center of the field by inspecting every tuber for damage by the pest. We then plotted the IPM knowledge of farmers vs. a pest control index calculated on the field pest infestation level.

*3) Efficiency of IPM information diffusion between graduate and exposed agents.* To measure the magnitudes of IPM learning spillovers from graduate to exposed farmers, we compiled data collected from farmer field school (FFS) events involving a total of 64 farmers conducted in a total of 5 communities [2, 3]. FFS is a form of adult education based on the concept that farmers learn optimally from field observation and experimentation. It was developed to help farmers tailor their IPM practices to changing ecological conditions. In regular sessions, groups of neighboring farmers perform simple experimentation which helps them further improve their understanding crop ecosystem functioning. In this learning process, farmers develop the expertise that enables them to make their own crop management decisions. Special group activities encourage learning from peers, and strengthen communicative skills and group building [24].

The methodology (described in detail in the references mentioned above) consisted in training 10-17 farmers in each community on IPM for *T. solanivora*. Both theoretical and practical training sessions were performed weekly or biweekly based on farmers’ convenience during at least three months. At the end of the sessions, all FFS graduates were encouraged to share their knowledge and learning experience with other farmers within their community. IPM knowledge about *T. solanivora* was assessed using a 20-item questionnaire 1) with community members before the FFS (control), 2) with graduate farmers after the FFS, and 3) with exposed farmers after the FFS.

**2. MODEL DESCRIPTION**

The model description follows the ODD (Overview, Design concepts, and Details) protocol for describing individual- and agent-based models [4-6] and consists of three elements. The first element provides an overview of the purpose, state variables and scheduling of the model. The second element explains general concepts underlying model design, and the remaining element provides details. The structure of the ODD protocol implies that redundancy can occur between the main document and the supporting information. The model was developed using CORMAS software (http://cormas.cirad.fr/en/outil/outil.htm).

2.1 Overview

*2.1.1. Purpose*

The model is a combined cellular automaton (CA) (SimPolilla, see Fig. S1 and [7]) and agent-based model (ABM) designed to explore the consequences of farmer’s agricultural practices on insect pest infestation levels during an IPM extension program. In this paper, the model was used in the specific scope of exploring the role of cooperative behaviours among small-scale farmers to manage an invasive potato tuber moth *Tecia solanivora*, Povolny (Lepidoptera: Gelechiidae) in the Ecuadorian Andes. The model simulates information diffusion, cooperation cost and effect, system sustainability (pest control) and was used to explore effective strategies to increase food security in the Andean region.

*2.1.2. State variables and scales*

The model comprises two hierarchical levels: the environment and the agents. The environment incorporates the thermal characteristics of the agricultural landscape and the population dynamics of the pest (Fig. 1 of the main document). It is modeled through a CA of 6 per 6 cells. Each cell of the CA represents a 500 per 500 m area, the total area corresponding to the size of a community (as defined below). Elemental cells do not represent individual potato fields but a territory, managed by single a group of farmers, in which the pest evolves. The spatio-temporal dynamics of *T. solanivora* (the pest) was driven by local environmental temperature and agents’ actions. Pest infestation level was simulated in each cell of the CA.

The agents’ level is represented by groups of potato growers. Various groups compose a community, i.e. groups of people living in the same locality and sharing common interests. A community is a clearly defined territory commonly referred as a village. In the Northern Andes, communities represent also a social and juridical network among people sharing the territory. In our model, each community contains 36 cells so that the total area is 900 ha, within the range of community sizes reported in central Ecuador [8]. In each community, we randomly placed 6 agents each one provided with a fixed land of 6 cells (Fig. 1). One agent represents a group of about 4 to 5 families of potato growers characterized by the same action taking rules and IPM knowledge, therefore applying similar agricultural practices in their fields. This is a common feature of the social organization in agricultural landscapes of the Ecuadorian Andes [9, 10]. If we assume that each family comprises 8 people, the density of inhabitants in our model is about 30 hab/km^2^, in the range of population densities found in the study region (Municipal Government of Guaranda, Bolivar, Ecuador). Each land corresponds to the territory an agent manages, and either represents his own land or one attributed to him by the community.

In our model, cooperation among agents occurred through the diffusion of IPM information from a trained agent through a FFS (named “graduate agent”) to other agents (named “exposed agents”) [11]. Agents were characterized by the following state variables: 1) their location within their community, 2) their IPM knowledge, 3) the number of actions (related to potato farming) they can do during one model step (named ‘time credits’ in the rest of the text), 4) their level of cooperation with other agents and 5) their social network (communication range) (Table S1).

We initiated model simulations with one graduate agent trained during a FFS and then simulated information diffusion through the community. Other agents had a IPM knowledge based on an empirical survey from farmer communities (see section 2. Empirical data). We defined a level of cooperation as the variable driving the willingness to diffuse of information through the community. Information diffusion occurred only on a fixed range of cells defined by the communication range state variable. As agents represent groups of farmers, this state variable introduces randomness in the probability of occurrence of a training session between two agents. All the parameters used in the model are summarized in Table 1. Each time step in the ABM corresponded to one pest generation (about two months).

*2.1.3. Process overview and scheduling*

The pest infestation level in each cell was driven by a CA (SimPolilla), whose conceptual description of processes and scheduling is provided in [7]. Briefly, it models the spatial temporal dynamics of the tuber moth *T.* *solanivora* in the Andes. Survival and reproduction are temperature dependent and dispersion through the landscape occurs through density-dependent diffusion.

Depending on their state variables values and pest infestation level in their field, each agent can make choices among alternatives behaviors with an associated cost in terms of time credits: 1) to be trained by a graduate agent about IPM practices, 2) to train an exposed agent about IPM practices, 3) to control the pest. Agents move randomly within their land after each action performed. Time is modeled as discrete steps and state variables are updated at each agent’s action within a time step.

2.2. Design concepts

*2.2.1. Emergence*

Pest infestation level included density-dependent dispersion and temperature-dependent survival and fecundity. Pest infestation level was also influenced by agents through IPM-knowledge based control so that pest population dynamics emerges from both the CA variables and agents’ behavior.

*2.2.2. Adaptation*

Agents make decisions depending on their IPM knowledge, their pest infestation level, their social network (other agents in their communication range), their remaining time credits, and their level of cooperation.

*2.2.3 Objective*

Agents’ main objective is to decrease pest infestation level on their land, thereby implicitly increasing their crop production and incomes.

*2.2.4. Learning*

As agents learn from others, they increase their IPM knowledge and pest control at two levels: 1) in their own fields and 2) in the whole community as they can train other agents to better control pest on their land.

2.2.5. *Prediction*

The tacit prediction of our model is that by training exposed agents, graduate agents would reduce pest infestation events from neighboring lands, therefore meeting their main objective.

*2.2.6. Sensing*

Agents perceive pest infestation level on their land. They also perceive both location and IPM knowledge of other agents in the community. We assumed that an agent *A_1_* can perceive the IPM knowledge of an agent A*_2_* thanks to observation of the level of pest infestation on A*_2_*’s land.

*2.2.7. Interaction*

Within a community, agents interact directly through informal IPM training sessions. Interactions are local, i.e. within the same community and within a range of 5 cells between agents (see hereafter for a discussion on communication range).

*2.2.8. Stochastic factors*

Stochastic factors are included in two sub-models. First, potato moth population dynamics is driven by stochastic temperatures within the range of those observed in the region [12]. Second, agents move stochastically within their land. Random numbers are generated using the VisualWorks #Random function (VisualWorks 7.5 Cincom Systems). Sensitivity analyses based on 100 replications over 120 time steps allowed us to analyze the outcome of the multiple sources of stochasticity resulting from the combination of our different sub-models.

*2.2.9. Observation*

Two types of data were collected from our model: IPM knowledge of agents and pest infestation levels. These data were collected at each time step and stored into external files.

*2.3. Details*

*2.3.1. Initialization*

*a. Level of pest infestation*

The initial level of pest in the grid cell was fixed to 1, the carrying capacity.

*b. Agent location*

Agents were randomly located on one of the 6 cells composing their land.

c. *IPM knowledge*

Following [13], we define knowledge as “*the possession of analytical skills, critical thinking, ability to make better decisions, familiarity with specific agricultural practices, and understanding of interactions within the agro-ecological system*”. Here, we focus on agent knowledge regarding IPM, and particularly potato moth control (see [14] for details on potato moth control practices).

To set up the IPM knowledge *X_a_* of each agent in the community, we used empirical data obtained from through interviews (see Appendix S1) during field surveys. As most IPM knowledge and practices for potato moth control are relatively easy to understand (e.g., keep the storage clean, store tuber in appropriate sacks), farmers can potentially answer correctly to all items of the interviews. Note, however, that there is often a limitation in the application of this knowledge, leading to limitation in IPM practices. This is actually shown by our survey of IPM knowledge levels within farmer communities (Fig. 2C) where no farmers with IPM knowledge = 5 were found. However, after an IPM session through farmer field school, we did observe that the level of knowledge can increase up to 5 (Fig. 2B). Based on these field data, we considered that the baseline proportion of the IPM knowledge scores (0, 1, 2, 3, 4, and 5) among agents was 1/6, 1/3, 1/3, 1/6, 0, and 0, respectively (Fig. 2A). We then simulated a scenario of IPM information spread where one agent has been trained through a FFS (IPM knowledge set to 5, see Fig. 2B) and potentially train the other agents in the community. The initial location of the graduate agent did not influence the model outputs for both IPM knowledge and pest infestation (see sensibility analysis performed by testing each potential location with 100 repetitions per location, Fig. S2).

*d. Communication range*

Fixing the value of communication range among agents was difficult due to the lack of empirical data. Consequently, we performed a sensitivity analysis to examine how communication range among agents, *ψ*, would influence pest infestation level dynamics in the community (Fig. S3). Results showed that for *ψ* > 3 (i.e. 1.5 km) the dynamics of pest infestation was not affected. Field observations and discussions with farmers revealed that farmers commonly communicate and cooperate with farmers living to distances greater than 1.5 km. The communication range among agents was set to *ψ* = 5 cells. As mentioned above, agents represent groups of farmers so that communication range introduces randomness in the probability of occurrence of a teaching session between two agents.

e*. Actions and time credits*

In our model, agents can perform two types of actions related to potato farming: 1) to conduct pest control applications (whose efficiency depends on their IPM knowledge) and 2) to exchange IPM information (either by being trained or training) so that they can increase their own IPM knowledge or that of other agents. These actions require time so that we included in our model two time credits parameters, *θ_training_* and *θ_control_*, for each type of action. We assumed that the total time *θ_total_* farmers dedicate to potato farming was:

$\theta_{total}=\sum(\theta_{training}+\theta_{control})$ Eq. 1

To define the different *θ* values we first arbitrarily set up *θ_total_* = 5 for each time step of the model. Then, following [3], who proposed that Andean farmers would accept to dedicate up to 10% of their total time (i.e. about 40% of their potato growing time) to IPM training sessions, we fixed *θ_training_* = 2. Finally, because IPM training is a time consuming process [13], we assumed (by default) that twice more time was needed to train than to control and fixed *θ_control_* = 1. The three potential combinations to use 5 units were: 1) 5 pest control actions, 2) 3 pest control actions + 1 training action, 3) 2 training actions and 1 pest control action. As one time step corresponds to 5 credits agents can do both training and pest control in each time step.

We performed a sensitivity analysis to explore how *θ_training_* values would influence pest infestation level dynamics (Fig. S4). The dynamics of pest infestation levels was similar among the four simulations, except in a short time frame between 4 and 7 years where infestation levels were slightly higher for lower *θ_training_* (maximum 10% after 5 years).

*2.3.2. Input data*

The stochastic factor used to modify temperature in each cell of the model was based on temperature variations measured in the study region (see the data in appendix 1 in [12]). Our model does not use other input data to represent time-varying processes.

*2.3.3. Sub-models*

*a. Potato moth population dynamics*

Details concerning this sub-model are described in SimPolilla (see [7] for a full description, validation, and sensitivity analyses).

*b. Potato moth control by agents*

Our field survey revealed that potato moth control increased linearly with increasing IPM knowledge of farmers (R² = 0.51, intercept *d* = 0.21, and slope *c* = 0.15; P < 0.001; see Fig 2C in the main document). Consequently, in our model, moth control Δ*_i_* by an agent *a* with an IPM knowledge *Χ_a_* was calculated as follows:

$\Delta_{i}=cX_{a}+d+\sigma$ Eq. 2

With *σ* the variance of pest control for a given IPM knowledge score given by box-plots in Fig. 2C in the main document.

*c. IPM information diffusion*

In our model, the decision of agents whether or not to share information (and their consequence on pest infestation levels) is included at three different levels. First, agent’s decision is explicitly calculated as a function of the level of pest infestation in their own fields, itself dependent on their IPM level of knowledge. As evidence by our field surveys, IPM level of knowledge level is closely related to farmer’s financial resources and personal situation within the community (see Dangles et al. 2010). Second, IPM diffusion process followed rules obtained from real-world data on the extent and the efficiency with which farmers share their knowledge (see Fig. 2B). We were indeed aware that many factors, including the social and economic status of farmers, may influence their decisions. However, obtaining reliable field data allowing establishing direct relationships between these factors and farmers’ IPM behavior is a challenging issue which has long been recognized by IPM extension program worldwide [25]. We therefore assumed that the real-world data of knowledge sharing included in our model represented the outcome of a decision making process made by farmers, including all sorts of important factors generally included in ABM such as information about incentives, payoffs, constraints such as economic cost, and uncertainty about the outcome. For example, if a farmer share the information with only 1 neighboring farmer, this could be due to various reasons such as 1) he has no incentive to share it (no problem with pest control), 2) he has a low social position in the community, or 3) limited financial resources make him dedicating his time to activities resulting in direct economical benefits (e.g., crop management, market). Third, we used our ABM to explore the relative benefits of IPM information sharing for pest control by explicitly comparing outcomes of simulations based on real-world diffusion process vs. a theoretical situation in which agents would not share information at all (see Fig. 5).

Technically, IPM information diffusion between two agents could occurred if the following conditions were fulfilled: 1) both agents should have different IPM knowledge (the agent with higher knowledge trained the other agent), 2) both agent should be located within a range of *ψ* = 5 cells from each other (communication range), and 3) both agents should have the necessary time credits left for a training IPM session (*θ_total_ ≥ θ_training_*). Agents preferentially assist neighbors, since this will increase their own potential pest protection. We considered that the efficiency of information diffusion was constant whatever the IPM knowledge scores of both graduate and exposed agents.

*d. Agents’ movement*

Agents moved randomly within their land after each action performed.

**3. Model analysis: verification and validation**

3.1. Verification

All sub-model of our ABM were tested using an external programming language [15] so that we verified that 1) the model was programmed correctly, 2) the algorithms were implemented properly, and 3) the model did not contain errors, oversights, or bugs. In addition to such routine verification, we also made verification that our model correctly reflects the workings of real-world process. In our case, one of the most important processes to reproduce was the relationship between IPM knowledge of farmers and pest control (see field data on Fig 2A of the main document). We did find that our model correctly reflected the relationships between IPM knowledge and pest control provided by field data (Fig. S5). No significant differences were found between the two regressions (ANCOVA, F = 0.97, P = 0.654).

3.2. Validation

The process of how new products or innovations get adopted as the consequence of interactions between users and potential users is traditionally modelled using the Bass model [16]. This model has been widely used in marketing and management science [17, 18]. Because this model fits the data of almost all product introductions, we used it to validate our agent-based diffusion of information, ensuring that our model correctly reproduces observed patterns in the literature. One interpretation of the Bass model was that the time *t* from information training until adoption is assumed to have a probability distribution *N(t*), which can e expressed as follows:

N_t+1_ = N_t_ + p(m − N_t_) + qN_t_(m − N_t_) / m Eq. 3

where *p* represents the coefficient of external influence, *q* the coefficient of internal influence and *m* the number of trained agents.

Eq. 3 is a difference equation and its solution is:

N_t_ = m * (1 − e^−(p+q)t^) / (1 + (q/p)e^−(p+q)t^) Eq. 4

Bass curve fitting to our ABM output data and estimation of *p*, and *q*, were performed following [19] and [20].

3.3. Analysis of the influence of cooperation

Although recent studies have shown that humans can behave altruistically [21, 22], decision of poor farmers to cooperate in a context of crop protection is likely to be driven by self interest (in fact, survival) rather than altruism [23]. In addition, farmers are more likely to behave depending on problems they physically perceive on their land rather than on behalf of agenda set by externally based science and development initiatives [2]. For those reasons, we assumed that farmers would be more prone to cooperate in IPM information diffusion when they perceived that a pest represents a danger for their crop production. Consequently, varying levels of cooperation in our model were obtained by changing the “pest danger threshold” perceived by agents, that is to say the pest infestation level that triggers a control action: lower threshold levels generated higher cooperation levels among agents (increased frequency in training session) and vice versa. This allowed us to fully couple both social and pest dynamics sub-models in our assessment of the role of cooperation for the success of IPM programs. Results of this analysis are presented in the Fig. 5 of the main document.

**References**

1. Bourgeois R, Jésus F (2004) Participatory prospective analysis: exploring and anticipating challenges with stakeholders, eds Bourgeois R, Jésus F (CAPSA Monograph Number 46. UNESCAP-CAPSA, Bogor, Indonesia).

2. Dangles O, Carpio FC, Villares M, Yumisaca F, Liger B et al. (2010) Community-Based Participatory Research Helps Farmers and Scientists to Manage Invasive Pests in the Ecuadorian Andes. *Ambio* 39: 325-335.

3. Villares M (2008) Implementation of a farmer formation system in integrated potato tuber moths management in bolivar (implementación de un sistema de capacitación de agricultor en manejo integrado del complejo de polillas de la papa en la provincia de bolivar), (Technical University of Bolívar, Bolívar, Ecuador).

4. Grimm V, Berger U, DeAngelis DL, Polhill JG, Giske J et al. (2010) The ODD protocol: A review and first update. *Ecological Modelling* 221: 2760-2768.

5. Grimm V, Berger U, Bastiansen F, Eliassen S, Ginot V et al. (2006) A standard protocol for describing individual-based and agent-based models. *Ecological Modelling* 198: 115-126.

6. Grimm V, Railsback SF(2005) Individual-Based Modeling and Ecology (Princeton University Press. Princeton).

7. Rebaudo F, Crespo-Pérez V, Silvain JF, Dangles O, (2011). Agent-Based Modeling of Human-Induced Spread of Invasive Species in Agricultural Landscapes: Insights from the Potato Moth in Ecuador. *Journal of Artificial Societies and Social Simulation* 14:3.

8. Gondard P, Mazurek H (2001) in 30 years of agrarian reforms and colonization in Ecuador (1964-1994): spatial dynamics (30 años de reforma agraria y colonizacion en el Ecuador (1964-1994): dinamicas espaciales). Dinamicas territoriales: Ecuador, Bolivia, Peru, Venezuela, Estudio de Geografia, eds Gondard P, Leon JB.

9. Larme A (1993) Work, reproduction, and health in two Andean communities (Department of Puno, Peru). Monograph, (Department of Anthropology of the University of North Carolina, Chapel Hill) 194 p.

10. Porter M, Monrad K (2001) Ayni in the global village: building relationships of reciprocity through international service-learning. *Michigan Journal of Community Service Learning*, University of Pittsburg 8: 1.

11. Pumisacho M, Sherwood S (2002) in Potato crop in Ecuador (El cultivo de la papa en Ecuador) (INIAP and CIP).

12. Dangles O, Carpio C, Barragan AR, Zeddam J-L, Silvain JF (2008) Temperature as a key driver of ecological sorting among invasive pest species in the tropical andes. *Ecological Applications* 18: 1795-1809.

13. Feder G, Murgai R, Quizon J (2004) The acquisition and diffusion of knowledge: The case of pest management training in farmer field schools, Indonesia. *Journal of Agricultural Economics* 55: 217–239.

14. Pollet A, Barragan AR, Zeddam J-L, Lery X (2003) Tecia solanivora, a serious biological invasion of potato cultures in south America. *International Pest Control* 45: 139-144.

15. R Development Core Team (2010) R: A language and environment for statistical computing. R Foundation for Statistical Computing, Vienna, Austria. ISBN 3-900051-07-0, URL http://www.R-project.org/.

16. Bass FM (1969) A new product growth for model consumer durables. *Manage. Sci.* 15: 215-227.

17. Chance DM, Hillebrand E, Hilliard JE (2008) Pricing an Option on Revenue from an Innovation: An Application to Movie Box Office Revenue, *Manage. Sci.* 54: 1015-1028.

18. Sood A, James GM, Tellis GJ (2009) Functional Regression: A New Model for Predicting Market Penetration of New Products, *Marketing Science* 28: 36-51.

19. Cowpertwait PS, Metcalfe AV (2009) in Introductory time series with R, (Springer, pp .51-55).

20. Kuandykov L, Sokolov M (2010) Impact of social neighborhood on diffusion of innovation S-curve. *Decision Support Systems* 48: 531-535.

21. O’Gorman R, Henrich J, Vugt MV (2009) Constraining free riding in public goods games: designated solitary punishers can sustain human cooperation, *Proceedings of Royal Society-B* 276: 323-329.

22. Vollan B, Ostrom E (2010) Cooperation and the Commons, *Science* 330: 923-925.

23. Brush SB (2004) in Farmer’s bounty (New Haven: Yale University Press).

24. Pontius, J.C., R. Dilts & A. Bartlett (2002) From farmer field school to community IPM: Ten years of IPM training in Asia. RAP/2002/15, FAO Regional Office for Asia and the Pacific, Bangkok. 106 pp.

25. Peshin R, Dhawan AK (2008) in Integrated pest management: dissemination and impact, eds Peshin R, Dhawan AK (Springer) 550p.

**Figure and table legends**

**Fig. S1. Simplified representation of the pest model.**

**Fig. S2.** **Effect of initial spatial configuration on information diffusion**. Each plot A, B, C, D, E and F are located in the figure to match the model configuration. Each curve represents the average IPM knowledge of 100 simulations for one agent, with standard deviation in dots. The red curves represent the information diffusion when initial graduate agent is in position C (central) and the black curves when he is on position A (corner), showing no significant difference in the information diffusion process.

**Fig. S3.** **Sensitivity of pest infestation levels to variations in the communication range parameter** *ψ* (from 1 to 10 cells). Curves are the mean of 100 simulations during 120 pest generations (20 years).

**Fig. S4.** **Sensitivity of pest infestation levels at the community level (6 agents) to variations in the time credit parameter.** Figure S4A represents variations in training time credit parameter *θ_training_* (from 1 to 4 credits) and figure S4B variations in control time credit parameter *θ_control_* (from 1 to 5 credits). Simulations with *θ_training_* = 5 were omitted as no farmer would spend 100% of his time in training sessions. Curves are the mean of 100 simulations during 120 pest generations (20 years).

**Fig. S5**. **Comparison of the relationship between IPM knowledge and pest control** either using data observed (in dark grey) or data simulated by our ABM model (in light grey). Thin lines represent the 95% confidence intervals.

**Table S1**. **Model parameters**. *a* and *i* denote parameters related to agents and cells, respectively.


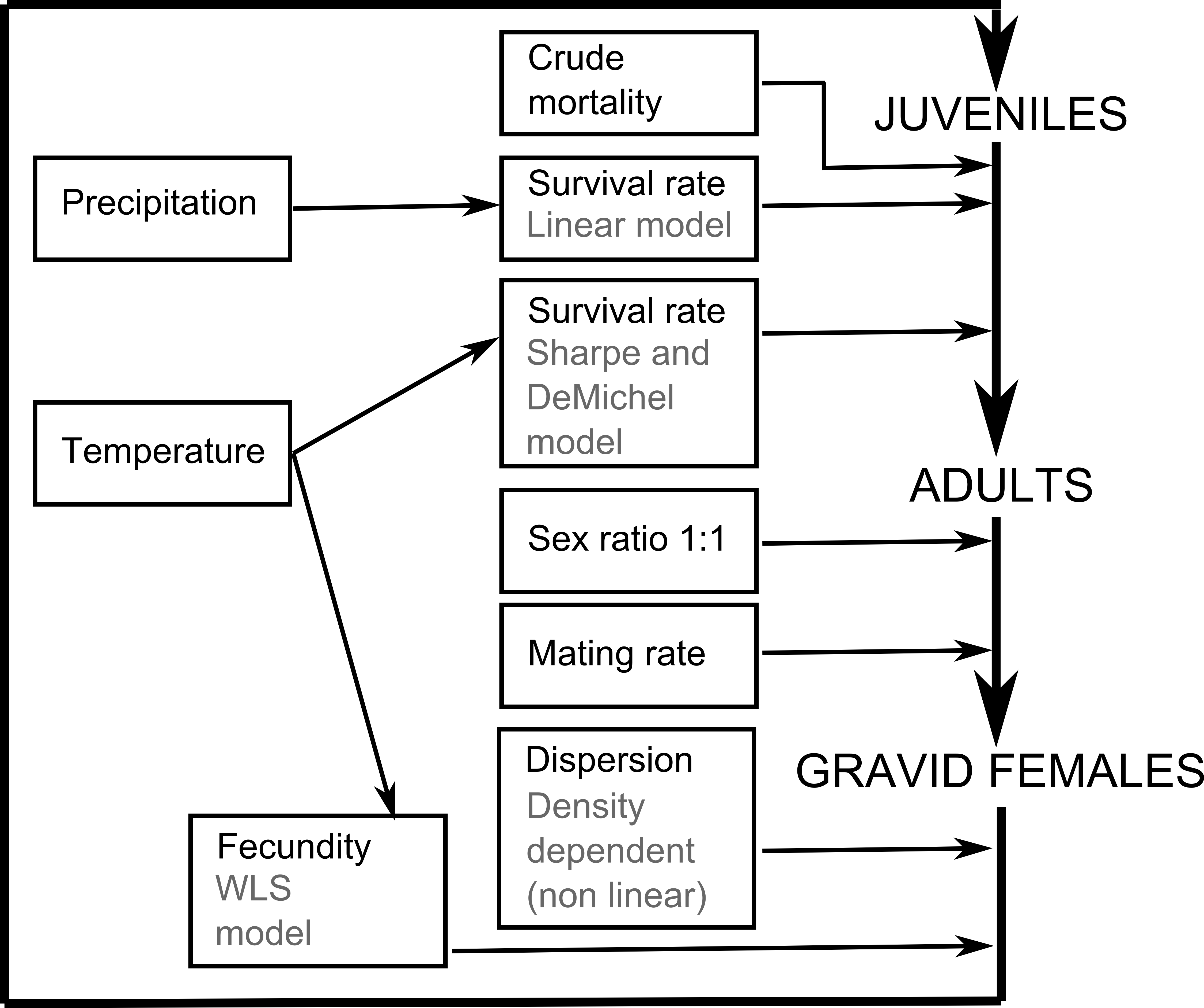


S1


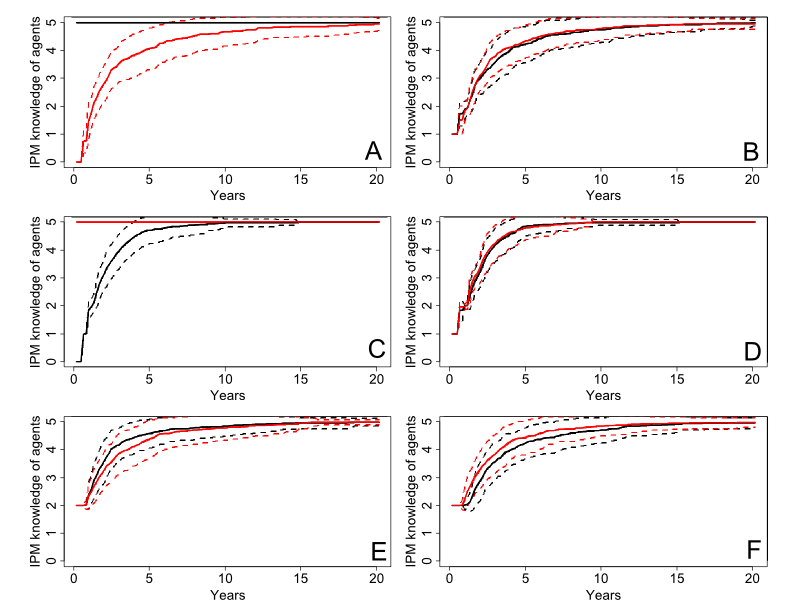


S2


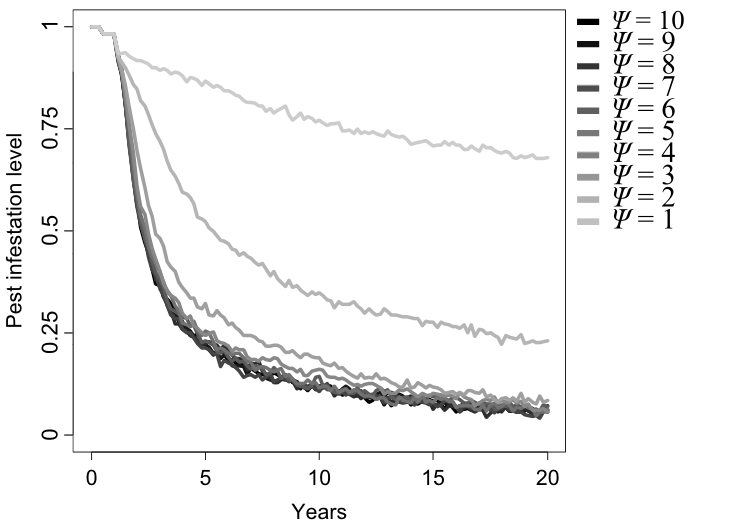
S3


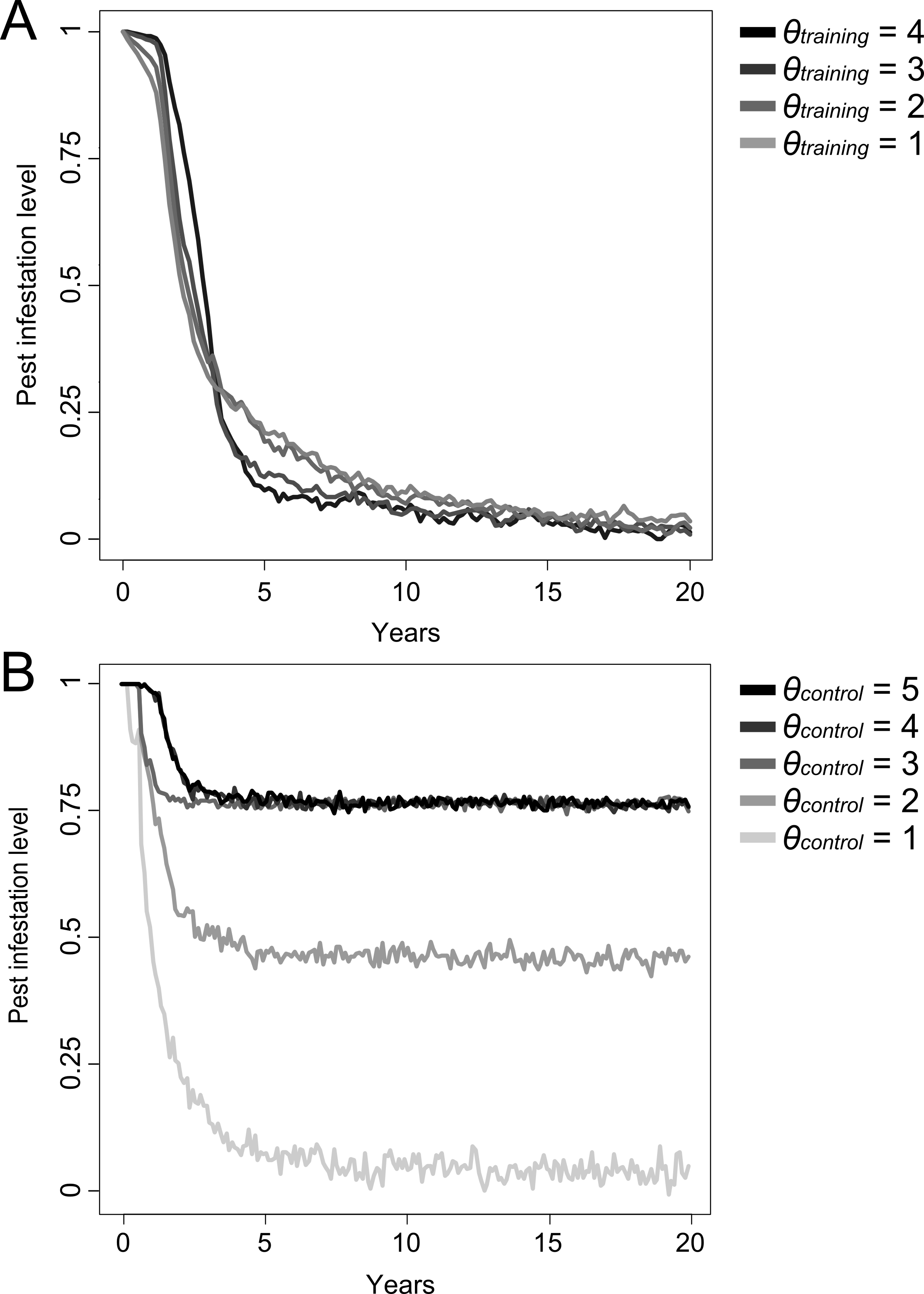


S4

**
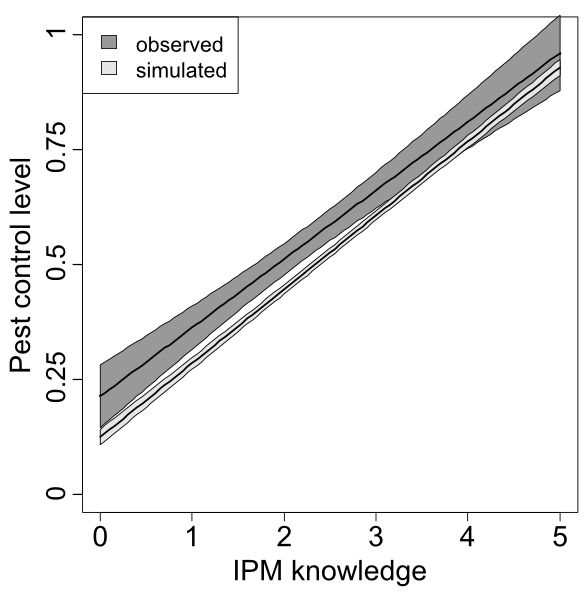
**

S5

| **Parameters** | **Name** | **Unit scale** | **Type** |
| --- | --- | --- | --- |
| IPM knowledge | *X_a_* | [0:5] | Field data |
| Moth control (auxiliary variable) | *Δ_i_* | [0:1] | Field data |
| Information diffusion efficiency | *Ε* | [0:1] | Field data |
| Pest infestation level | *G_i_* | [0:1] | CA output |
| Level of cooperation | *Κ_a_* | [0:1] | Variable |
| Time credits | *θ_a_* | 5 | Constant |
| Communication range | *Ψ* | 5 | Constant |

Table S1

**Appendix S1 – Questionnaire (in Spanish) use to measure the knowledge of farmers concerning the IPM of potato moths in Ecuador**

🞎 Hombre 🞎 Mujer Edad**: _ _ _ _ _ _ _ _ _ _ _ _** Comunidad: _ _ _ _ _ _ _ _ _ __

**1. ¿Conoce acerca de la Mariposa de la papa?**

🞎 Si 🞎 No

**2. ¿Como cree que llegó la polilla de la papa a su campo?**

🞎 Dentro de la semilla 🞎 Volando 🞎 Caminando 🞎 Otras …………………..

**3. Cuantos tipos de polilla de la papa conoce?** …………….

**4. Podría describir como son la/as polillas o mariposas de la papa que conoce?**

🞎 Con círculos en las alas 🞎 Con triángulos en las alas 🞎 Puntitos en las alas 🞎 Otras……

**5. Conoce cómo se reproduce la polilla de la papa? Explique**

🞎 No 🞎 Si……………………………...

**6. Describa los cambios que sufre la polilla de la papa durante su vida?**

🞎 Huevo, larva o gusano, adulto o mariposa

🞎 Huevo, larva o gusano, pupa o adivinador, adulto o mariposa

🞎 Larva o gusano, adulto o mariposa

🞎 Otra..........................................................................................................

**7. En que estado de la vida de la polilla produce daños al tubérculo?**

🞎 Huevo 🞎 Larva o gusano 🞎 Pupa o adivinador 🞎 Adulto o mariposa 🞎

**8. ¿Conoce usted a que parte de la planta de papa ataca la polilla?**

🞎 Hojas 🞎 Tallo 🞎 Tubérculo

**9. ¿En que parte o lugar a visto a los adultos o mariposas de la polilla de la papa?**

🞎 Base de las plantas 🞎 Hojas 🞎 Bodega

**10. ¿Según Usted en que condiciones climáticas aumenta más la polilla?**

🞎 Calor 🞎 Frío 🞎 Lluvia 🞎 Viento

**11. ¿Se puede sembrar la semilla si esta picada?**

🞎 Si 🞎 A veces 🞎 Trato de no hacerlo 🞎 Nunca

**12. ¿Que rotación de cultivos usted hace?**

🞎 No roto 🞎 Papa / pasto 🞎 Papa / otros cultivos: _ _ _ _ _ _ _ _

**13. Después de haber preparado el suelo:**

🞎 Siembro inmediatamente para ganar tiempo

🞎 Dejo pasar unos días: ¿cuantos días? _ _ _ _ _ _ _ _ _ _ _ _

**14. ¿Usted hace aporque en su cultivo de papa?**

🞎 No

🞎 Si: 🞎 Aporque normal 🞎 Aporque alto

**15. ¿Una vez que las plantas han madurado, usted corta el follaje?**

🞎 No, dejo que se marchite en el campo 🞎 A veces

🞎 Si: ¿Que hace con este follaje?

🞎 Lo dejo en el cultivo 🞎 Lo boto en una quebrada 🞎 Lo quemo

🞎 Lo utilizo como comida para el ganado

**16. ¿En el caso que tenga papa picada o poco picada, que usted hace?**

🞎 La dejo en el cultivo 🞎 La doy a los chanchos

🞎 La boto en una quebrada 🞎 La entierro profundo

**17. ¿Cómo usted guarda la papa cosechada?**

🞎 Amontonada

🞎 En sacos: 🞎 normales 🞎 ralos

**18. ¿Usted realiza limpieza del almacén antes de guardar las papas?**

🞎 No 🞎 Si 🞎 A veces

**19. ¿Usted fumiga el almacén?**

🞎 No 🞎 Si 🞎 A veces

**20. ¿Usted utiliza plantas como Eucalipto o Marco como repelentes?**

🞎 No 🞎 Si 🞎 A veces
